# Techniques and long-term effects of transjugular intrahepatic portosystemic shunt on liver cirrhosis-related thrombotic total occlusion of main portal vein

**DOI:** 10.1038/s41598-017-11455-y

**Published:** 2017-09-07

**Authors:** Lei Wang, Fuliang He, Zhendong Yue, Hongwei Zhao, Zhenhua Fan, Mengfei Zhao, Bin Qiu, Jiannan Yao, Qiushi Lin, Xiaoqun Dong, Fuquan Liu

**Affiliations:** 1 Department of Interventional Therapy, Beijing Shijitan Hospital, Capital Medical University, Beijing, P. R. China; 2 Beijing Chaoyang Hospital, Capital Medical University, Beijing, P. R. China; 3 Department of Medicine, Warren Alpert Medical School of Brown University, Rhode Island, USA

## Abstract

Portal vein hypertension (PVH) in liver cirrhosis complicated with portal venous thrombosis (PVT) has been mainly treated with transjugular intrahepatic portosystemic shunt (TIPS). The clinical effects of TIPS have been confirmed, however, no large-scale studies have been focused on technical analyses and a long-term follow-up, especially on thrombotic total occlusion of main portal vein (MPV). To demonstrate critical techniques and clinical outcome of TIPS on liver cirrhosis-related thrombotic total occlusion of MPV, 98 patients diagnosed with liver cirrhosis related thrombotic total occlusion of MPV and treated with TIPS from January 2000 to January 2010 were retrospectively analyzed. Twenty-three (23.5%) patients had MPV (single site) thrombosis, 55 (56.1%) had multiple site-thrombosis (MPV and other), 17 (17.3%) had cavernous transformation of portal vein, and 3 (3.1%) had post-transplant thrombosis. The successful rate of TIPS was 90.7%, without any procedure-related deaths or severe complications. Mean portal pressure was dropped from 33.08 ± 1.38 mmHg preoperatively to 20.18 ± 0.83 mmHg postoperatively (*p* < 0.001). Collectively, TIPS is safe and effective in treating liver cirrhosis-related thrombotic total occlusion of MPV. This complex procedure requires combination of indirect portography and percutaneous transhepatic portal techniques to increase the rate of success.

## Introduction

Portal vein thrombosis (PVT) is one of the most common complications of liver cirrhosis, with an incidence of 1–32%^1–14^. PVT has been treated with various methods^15–17^ such as anticoagulants, surgery, and interventional therapy depending on acute or chronic onset, anatomical region, grade of thrombosis, and clinical symptoms.

With the development of techniques in the past more than two decades, transjugular intrahepatic portosystemic shunt (TIPS) has been widely applied. Its indications have been gradually expanded from acute and uncontrollable gastrointestinal bleeding, recurrent bleeding and refractory ascites to Budd-Chiari syndrome, hepatic hydrothorax, hepatopulmonary syndrome, preoperative prevention of surgical complications of liver cirrhosis, and even portal vein thrombosis^18–22^. In particular, TIPS has been converted from a contraindication to an indication of portal vein thrombosis, which is now regarded as a classic subversion in the application of TIPS. Recently, TIPS has become one of the key tools for treating portal vein thrombosis resulted from liver cirrhosis. The clinical effects of TIPS have been confirmed, however, there is no study conducted in a large-sample size, involving comprehensive technical exploration, and a long-term follow-up, especially focused on the prognostic value of TIPS on thrombotic total occlusion of main portal vein (MPV)^14, 23^.

To fill in gaps in knowledge, we recruited 98 patients with liver cirrhosis related thrombotic total occlusion of MPV. The patients were treated by TIPS at Beijing Shijitan Hospital of Capital Medical University.

## Materials and Methods

### Clinical subjects

From January 2000 to January 2010, 98 patients [67 men and 31 women; mean age of 44.8 (range 32–68) years] were randomly recruited. Those patients were diagnosed of liver cirrhosis complicated with thrombotic total occlusion of MPV, including 76 with hepatitis B cirrhosis, 4 with hepatitis C cirrhosis, 6 with alcoholic cirrhosis, 1 with primary biliary cirrhosis, 2 with autoimmune cirrhosis, 5 with Budd-Chiari syndrome, and 4 with cryptogenic cirrhosis. The preoperative Child-Pugh classification was “A” in 21.4% (21/98), “B” in 46.9% (46/98), and “C” in 31.6% (31/98) of the patients. The mean MELD score, bilirubin, ammonia, ALT, and INR was 10.2 ± 2.8, 30.34 ± 41.63 µmol/L, 62.55 ± 28.46 µmol/L, 42.66 ± 20.22 U/L, and 1.41 ± 0.28, respectively. Among them, 89 had gastrointestinal bleeding, 18 had refractory ascites/hydrothorax and ascites (9 patients had gastrointestinal bleeding), 5 had severe liver dysfunction, and 8 had enhanced thrombosis during therapy, as presented in Table 1. Besides MPV, total occlusion of other vessels was also detected, as shown in Table 2.

**Table 1:** Relationship between thrombotic types and clinical symptoms.

Anatomical occurrence sites	No. of patients	Percentage (%)	Gastrointestinal bleeding	Refractory ascites	Severe liver dysfunction	Increase in thrombus
P	23	23.5	22	3	0	2
pb	25	25.5	24	5	1	3
pbm	9	9.2	7	2	1	1
pbs	1	1.0	1	0	0	0
pbms	7	7.1	6	2	1	0
pm	9	9.2	8	1	1	1
ps	3	3.1	3	0	0	0
pms	1	1.0	1	0	0	0
pc	17	17.4	15	4	1	1
pl	3	3.1	2	1	0	0
Total	98	100	89	18	5	8

Severe liver dysfunction was defined as total bilirubin ≥ 50, ATP < 40%, and INR >1.5; or bilirubin >100. INR, international normalized ratio.

Increase in thrombus: an increase in the number of thrombus after treatment with other methods during a specific period (generally 2 weeks), or a gradual increase in the number of thrombus lasted for >1 week during the treatment.

MPV, main portal vein; pb, main portal vein + branch; pbm, main portal vein + branch + superior mesenteric vein; pbs, main portal vein + branch + splenic vein; pbms, main portal vein + branch + superior mesenteric vein + splenic vein; pm, main portal vein + superior mesenteric vein; ps, main portal vein + splenic vein; pms, main portal vein + superior mesenteric vein + splenic vein; pc, main portal vein + cavernous transformation; pl, main portal vein + liver transplantation.

**Table 2:** Relationship between thrombotic type and number of occluded vessel.

Anatomical occurrence site	Total number of patients	One occluded vessel	Two occluded vessels	≥3 Occluded vessels
p	23	23	0	0
pb	25	19	6	0
pbm	9	6	2	1
pbs	1	1	0	0
pbms	7	4	2	1
pm	9	2	5	2
ps	3	2	1	0
pms	1	0	0	1
pc	17	4	10	3
pl	3	2	1	0
Total	98	63	27	8

pb, main portal vein + branch; pbm, main portal vein + branch + superior mesenteric vein; pbs, main portal vein + branch + splenic vein; pbms, main portal vein + branch + superior mesenteric vein + splenic vein; pm, main portal vein + superior mesenteric vein; ps, main portal vein + splenic vein; pms, main portal vein + superior mesenteric vein + splenic vein; pc, main portal vein + cavernous transformation; pl, main portal vein + liver transplantation.

### Methods

#### Preoperative preparations

Clinical examinations including routine blood, urine, and stool, liver and renal function, coagulation function, blood type, electrocardiography, liver and splenic ultrasonography, hepatic vascular ultrasonography, abdominal enhanced computed tomography (CT), and/or magnetic resonance imaging (MRI), gastroscopy and/or upper gastrointestinal barium meal radiography were performed. For those patients with rare types of liver cirrhosis, special examinations were also performed to exclude bleeding due to gastrointestinal ulcer or other diseases. Liver function, coagulation function, and platelet count were adjusted if necessary to meet the indications for TIPS.

The patients and their relatives were informed about the potential outcomes and risks of TIPS procedure. A written consent to undergo TIPS and to participate in this research was signed by each subject. This manuscript contains no information or images that could lead to identification of a study participant. This study was approved by the Institutional Review Board of Beijing Shijitan Hospital and conducted in accordance with all current ethical guidelines.

#### Major procedures

**Figure 1:**
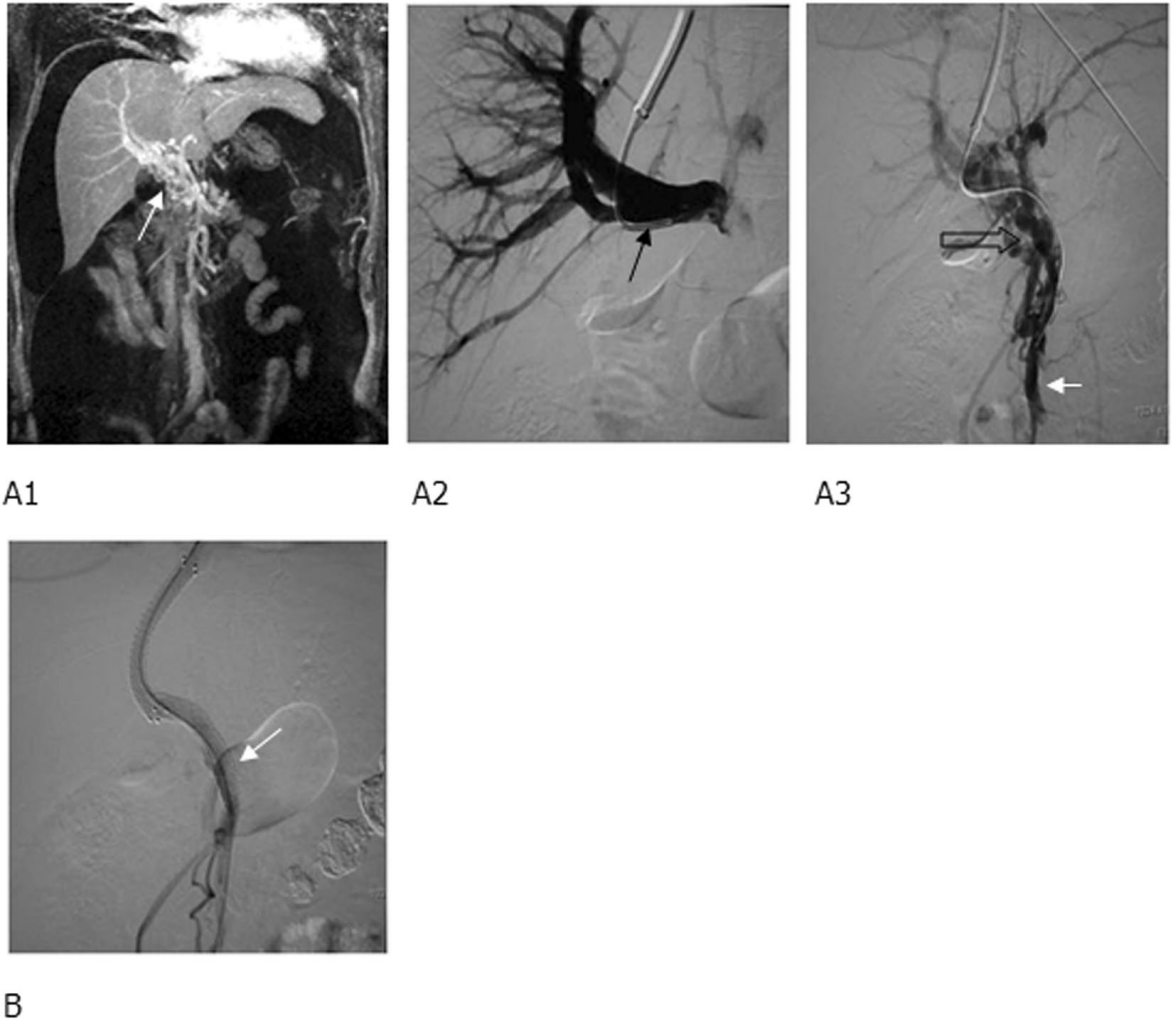
MRPV (Magnetic Resonance of portal vein) and TIPS (transjugular intrahepatic portosystemic shunt) portography showed (**A1**) cavernous transformation of portal vein (white arrow), right portal branch (black arrow), (**A2**) occluded trunk of superior mesenteric vein (hollow arrow), and (**A3**) a relatively large branch (short and white arrow). (**B**) Two BMS (bare metal stent) were implanted between a hepatic vein and a relatively large branch of superior mesenteric vein to form a shunt, and the shunting blood flow was smooth, with the disapperance of a collateral vessel (white arrow).

**Figure 2:**
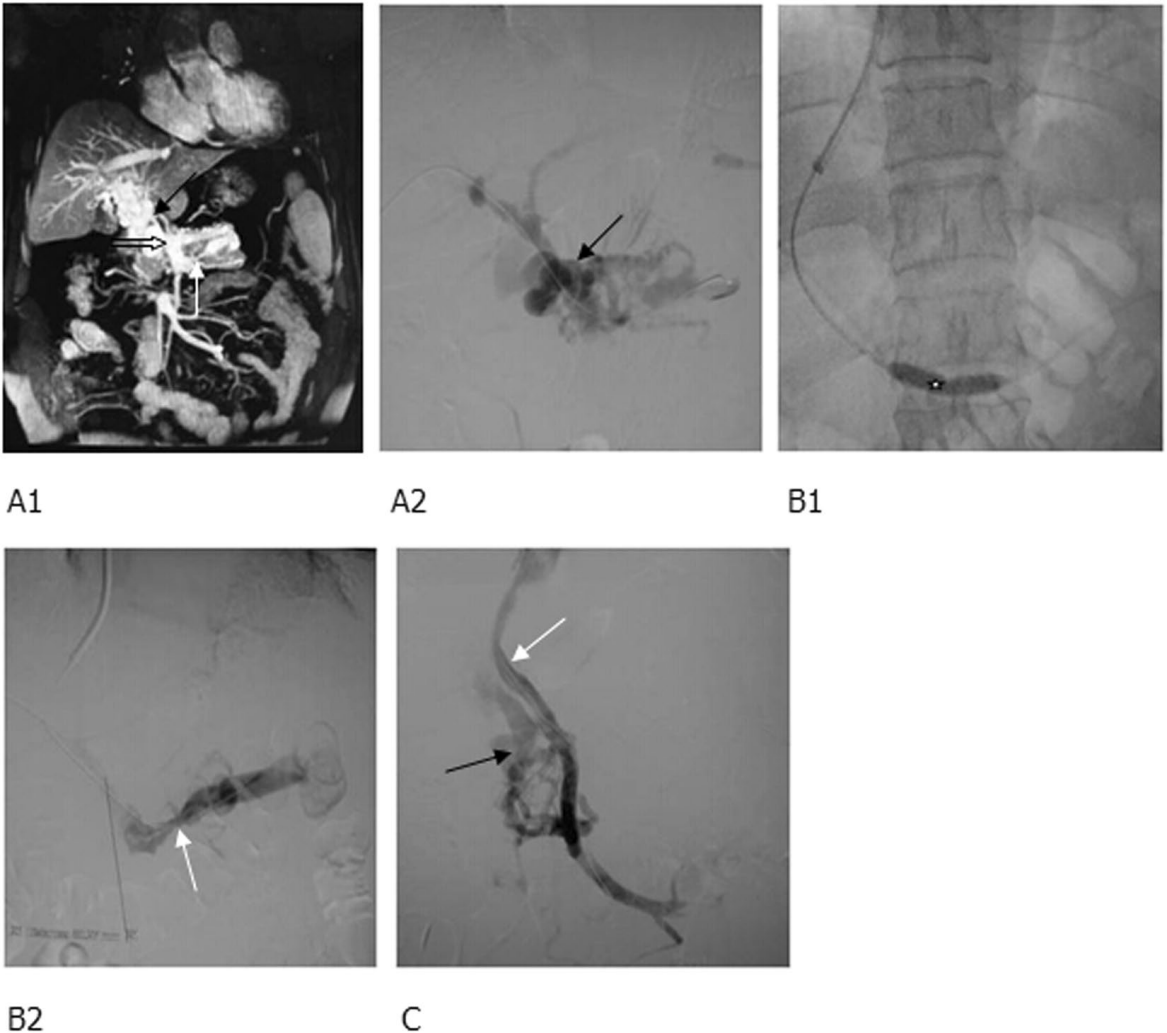
MRPV and percutaneous transhepatic portography showed (**A1**) cavernous transformation of portal vein, numerous collateral vessels (black arrow) and (**A2**) occlusion at the openings of superior mesenteric vein (white thick arrow) and splenic vein (white thin arrow). (**B1**) After balloon dilatation of the thrombi in the splenic vein (white star), the splenic vein was opened and the collateral vessels were markedly reduced, (**B2**) however, an obvious stenosis (white arrow) remained there. (**C**) The superior mesenteric vein was re-opened and 2 BMS were implanted to form a shunt of smooth blood flow (white arrow), and some collateral vessels were observed (black arrow).

For those with left and/or right portal branch thrombosis with filling defect on preoperative imaging, a catheter was placed through the femoral artery to perform indirect portography, followed by TIPS (Fig. 1). In brief, a catheter was placed through jugular vein to inferior vena cava or hepatic vein to puncture portal vein for portography, pressure measurement, portal vein embolization, balloon dilatation of shunt, and stent implantation to form a shunt. If failed, a 4/5 F imaging catheter was inserted percutaneously and transhepatically into the portal vein, so that we could puncture towards the catheter since it clearly demonstrated the course of portal vein. TIPS was then performed (Fig. 2).

**Figure 3:**
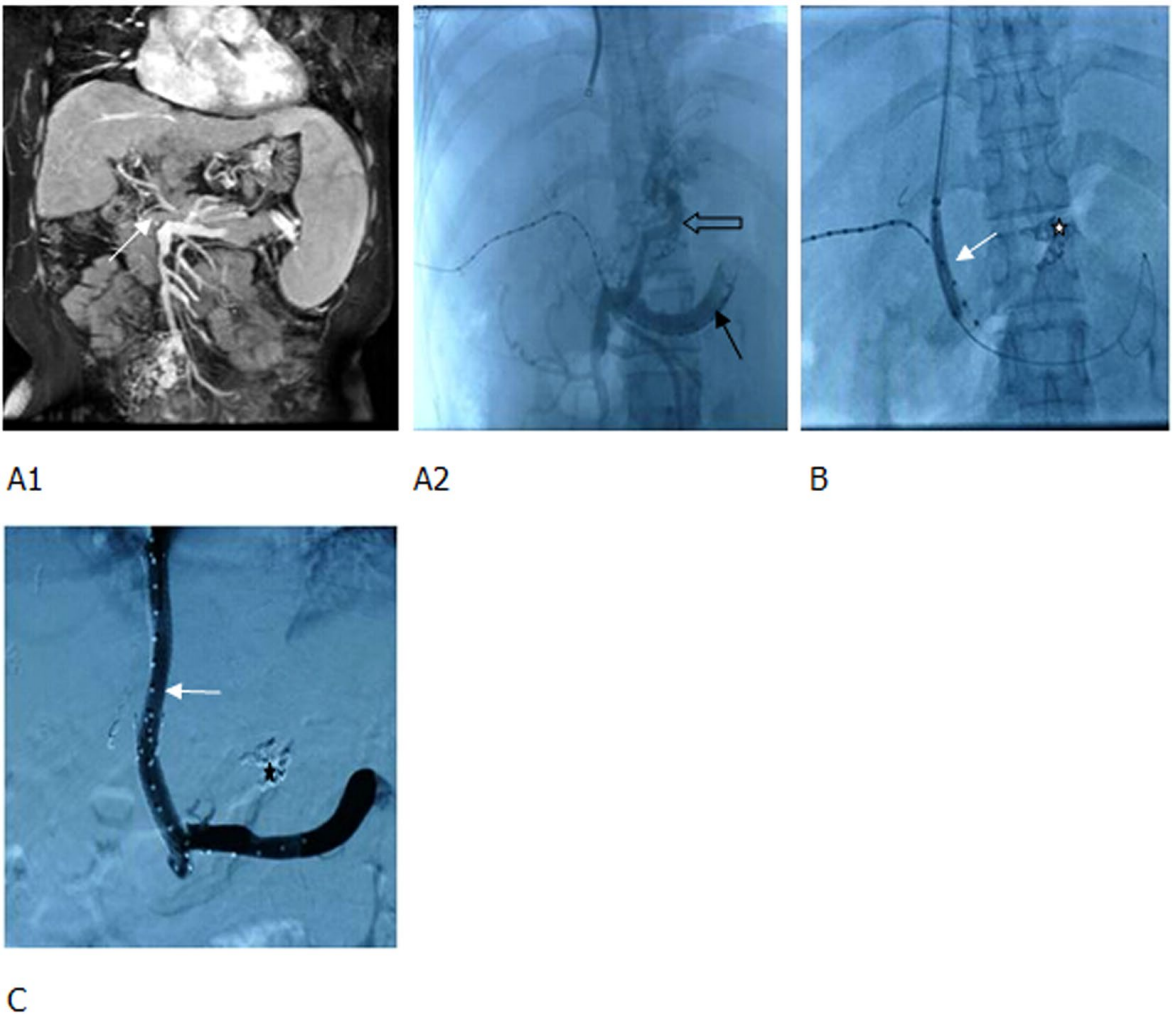
MRPV and percutaneous transhepatic portography showed (**A1**,**A2**) the thrombotic total occlusion of MPV (main portal vein) (white arrow), catheter penetrating through the thrombus to the splenic vein (black arrow), a significant varicose gastric coronary vein (hollow arrow), and countercurrent contrast agents in the superior mesenteric vein. (**B**) TIPS sheath was inserted into the MPV, a balloon was used to dilate the thrombus; shunt (white arrow) and coils were applied to embolize the gastric coronary vein (white star). (**C**) Two BMS were used to create a shunt. The blood flow in the shunt was smooth (white arrow) and the varicose vein disappeared (black star).

**Figure 4:**
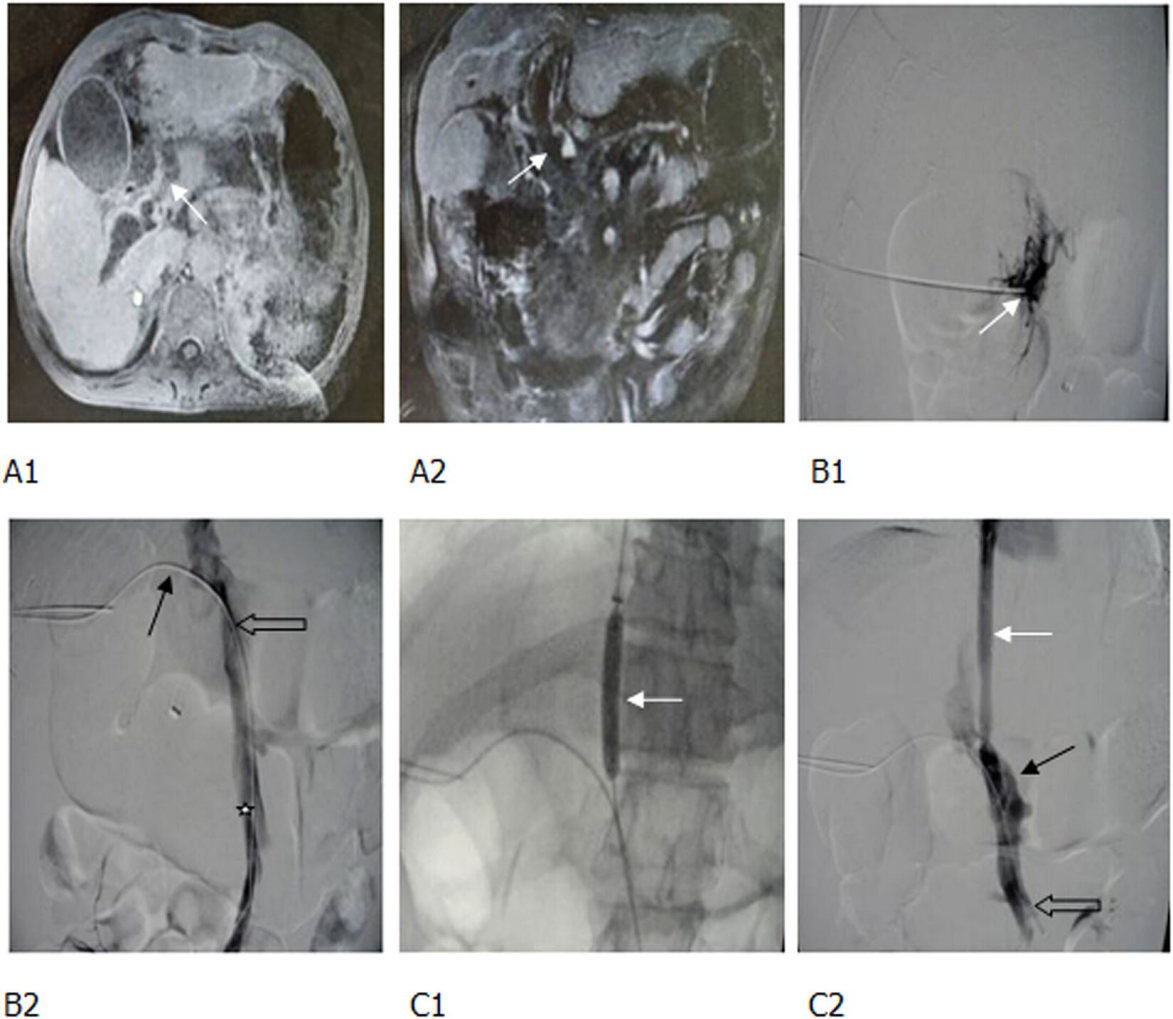
Thrombotic total occlusion of portal vein following splenectomy, gastrointestinal bleeding. (**A1**, **A2**) Enhanced MR and MRPV identified a totally occlusive thrombus in the MPV (white arrow). (**B1**, **B2**) The puncture needle was inserted into the thrombus following one-step procedure (white arrow), but it failed to pass through the thrombus. Therefore, a 18 G puncture needle was used, and the guide wire and catheter (black arrow) passed through the thrombus. Percutaneous transhepatic portography showed a totally occlusive thrombus in the MPV (hollow arrow) and partial thrombosis of superior mesenteric vein (white star). (**C1**, **C2**) The balloon-dilated thrombus and a shunt (white arrow). After implanting one covered stent to form a shunt, both the shunt (white arrow) and portal vein (black arrow) were unblocked. A few thrombi remained in the superior mesenteric vein (hollow arrow).

**Figure 5:**
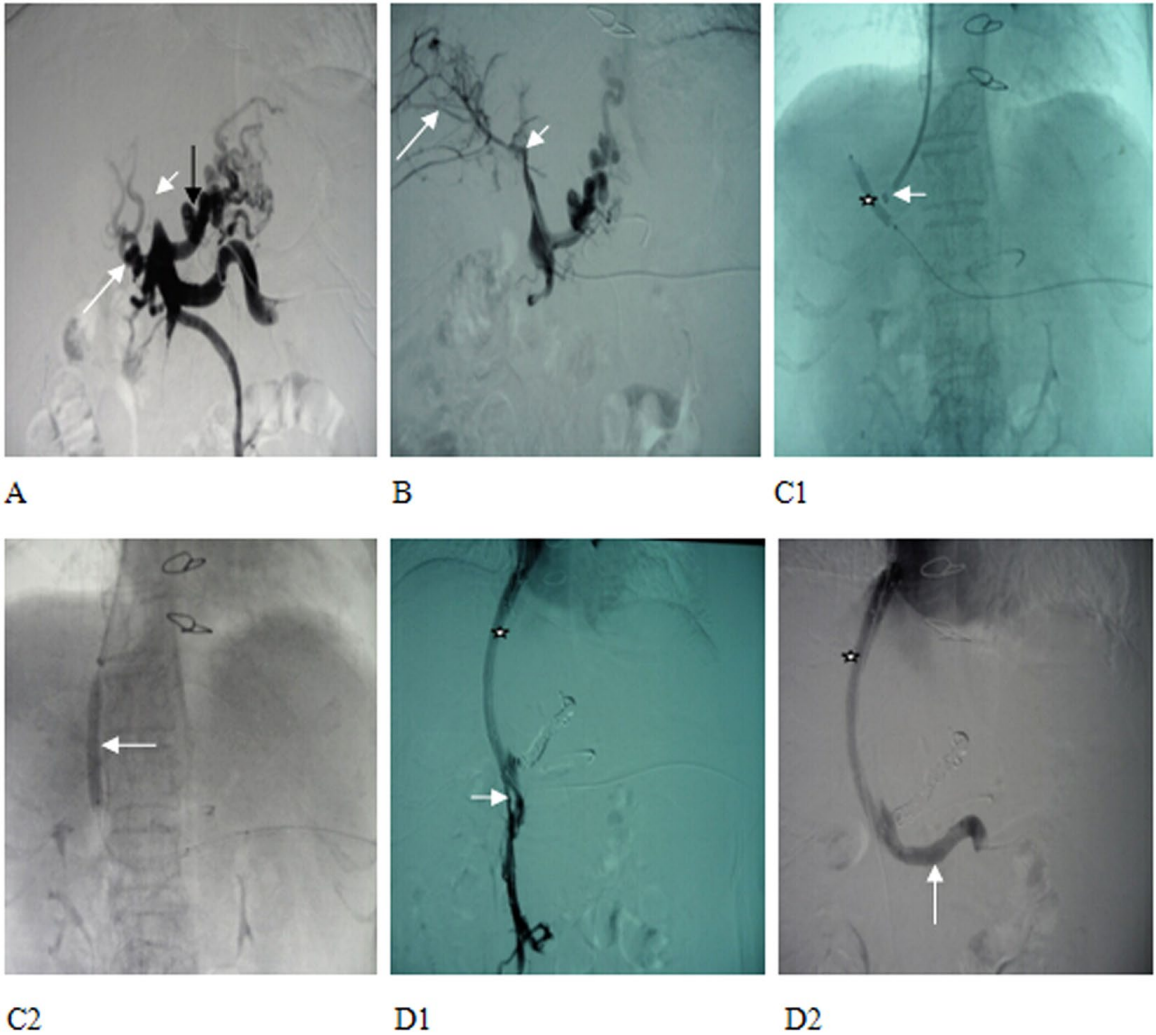
Thrombotic total occlusion of MPV (short and white arrow). (**A**) small amount of collateral formation was observed (long and white arrow), and gastric coronary vein presented with obvious varicoses (long and black arrow). (**B**) Splenic venous catheter highly selective for a branch of portal vein. Portography identified intrahepatic portal vein thrombosis (short and white arrow) and small branches (long and white arrow). (**C1**, **C2**). A balloon (6 × 40 mm) was used to dilate the thrombus (white star). The outer sheath of RUPS-100 (short and white arrow) alignment balloon punctured intrahepatic portal vein, a balloon (8 × 40 mm) was applied to dilate distributary channel (long and white arrow). (**D1**, **D2**) Portography identified the smooth blood flow in the shunt (white star) and superior mesenteric vein, where opening residual thrombosis (short and white arrow) had been opened. (long and white arrow) was unblocked without thrombus.

For those with intrahepatic left or right portal vein without filing defect on preoperative imaging, a percutaneous puncture was directly performed to place a catheter in the portal vein, followed by TIPS (Figs 3 and 4). If direct percutaneous transhepatic portal vein failed, modified percutaneous transsplenic portal vein puncture was used, and then TIPS procedure was performed (Fig. 5).

**Figure 6:**
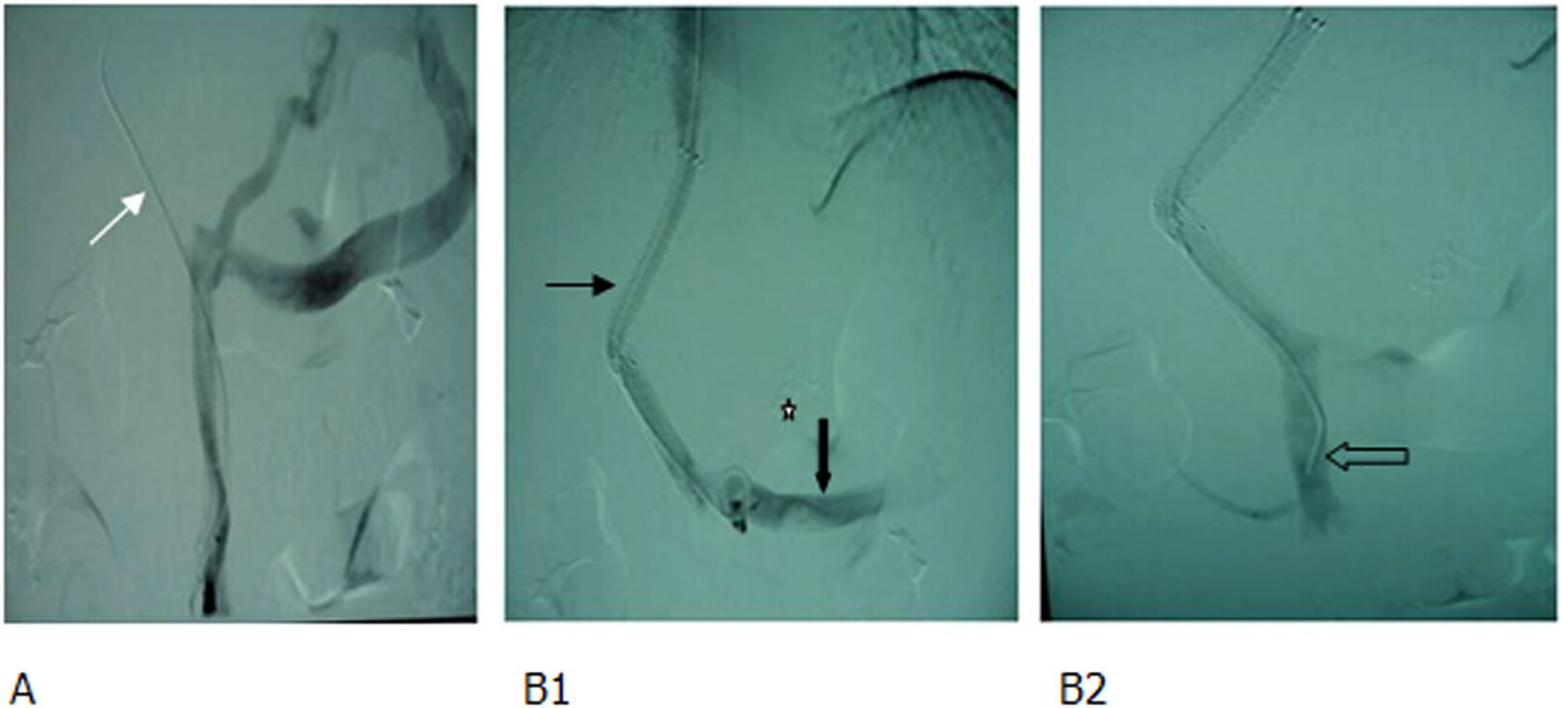
Acute total portal venous thrombosis in patients with liver cirrhosis and gastrointestinal bleeding. (**A**) Portography showed that the contrast agents was passing through the superior mesenteric vein to gastric coronary vein and retrograding to splenic vein, whlie the thrombus totally occluded portal vein (white arrow). (**B1**, **B2**) Portography showed that two BMS had formeda shunt (thin black arrow) while coils had embolized gastric coronary vein (white star). The shunt, superior mesenteric vein (hollow arrow) and splenic vein (thick black arrow) were unblocked.

**Figure 7:**
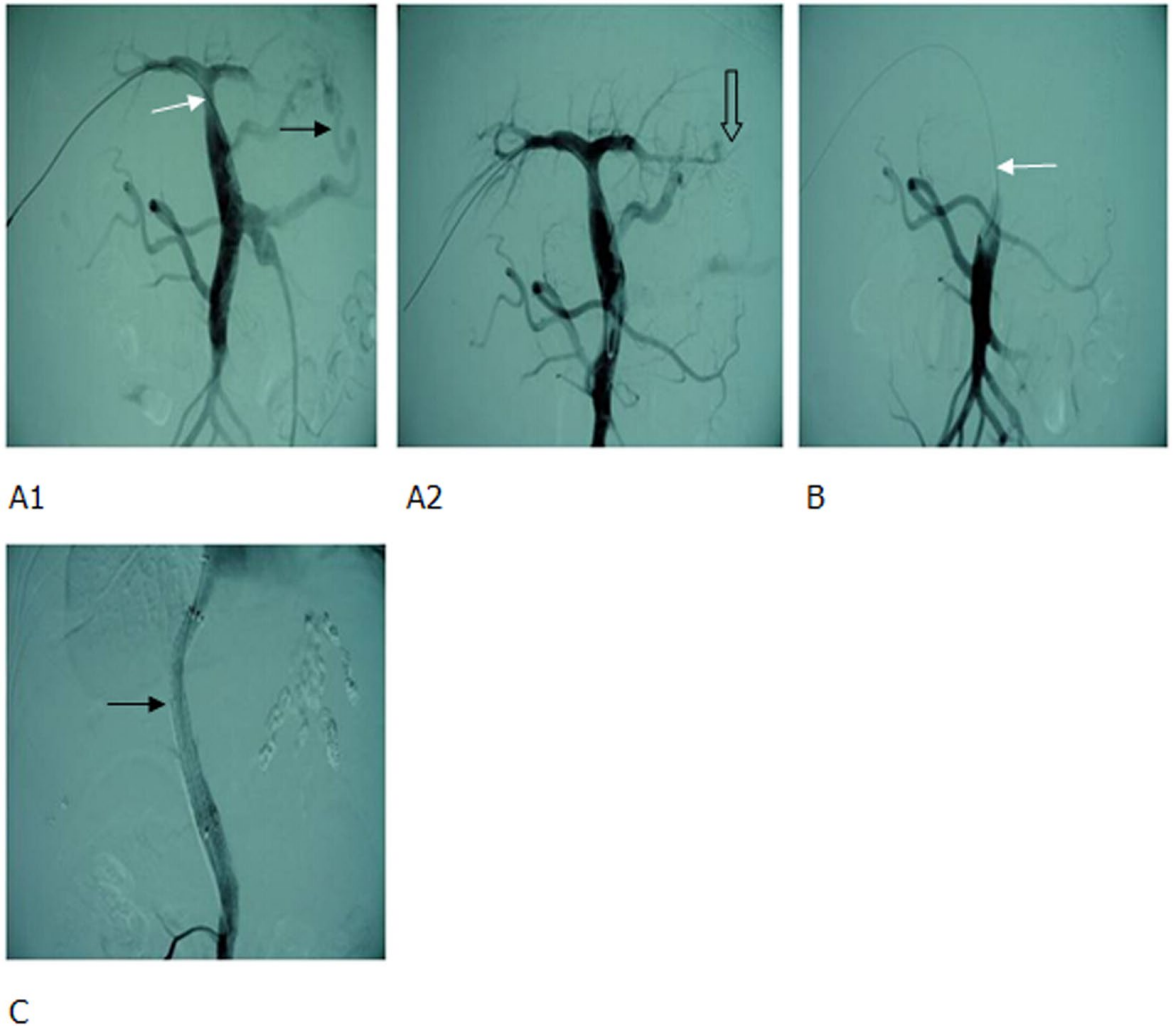
After cirrhotic liver transplantation, gastrointestinal bleeding, and total thrombosis of portal vein occurred. (**A1**, **A2**) Percutaneous transhepatic portography identified stenosis of the distal end of MPV (white arrow) and significant varicoses (black arrow). After embolization, varicoses (hollow arrow) disappeared. (**B**) At 2 months following varicocele embolization, total thrombus was observed in portal vein (white arrow). (**C**) During TIPS, 2 covered stents were implanted to creat a shunt (black arrow).

For those with acute portal thrombosis, TIPS was directly performed if the patients had combined gastrointestinal bleeding (Fig. 6). If it failed, a percutaneous puncture was performed to place a catheter in the portal vein, followed by TIPS (Fig. 7).

**Figure 8:**
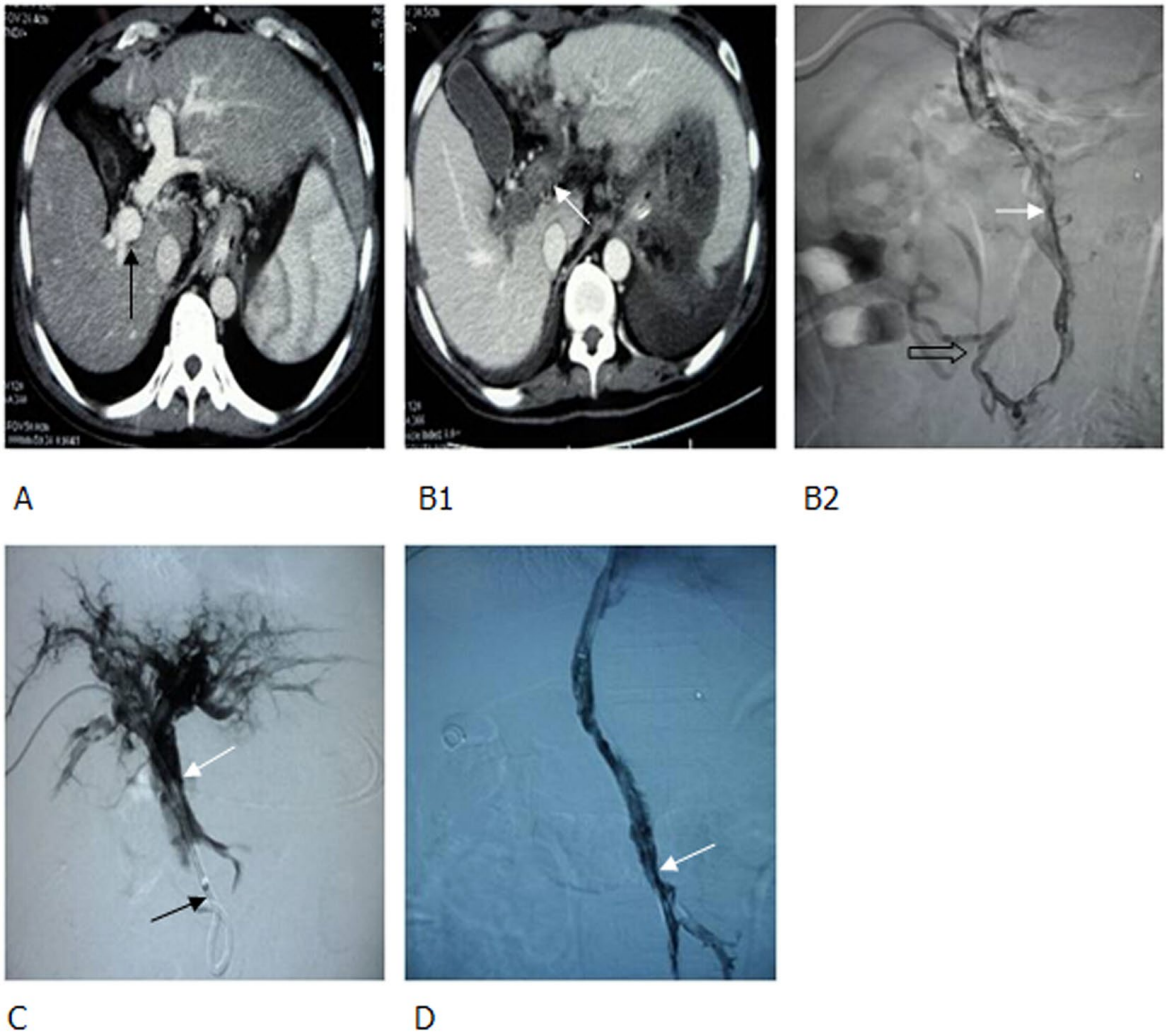
Cirrhotic portal hypertension and total thrombosis in portal vein 5 days after splenectomy, percutaneous transhepatic embolectomy and thrombectomy; 2 days after local thrombolysis and anticoagulant therapy, complicated with gastrointestinal bleeding. (**A**) Before splenectomy, enhanced CT scan demonstrated that portal vein was completely unblocked (black arrow), without any thrombus. (**B1**, **B2**) At the third and fifth day following splenectomy, enhanced CT scan and percutaneous transhepatic portography identified total thrombi in portal vein and superior mesenteric vein (white arrow). A few collateral vessels of superior mesenteric vein were present (hollow arrow). (**C**) After embolectomy, thrombectomy was conducted with a balloon, local thrombolysis, and anticoagulant therapy. Thus, thrombi in portal vein were decreased (white arrow), however, thrombi in superior mesenteric vein were not significantly changed (black arrow), complicated with gastrointestinal bleeding. (**D**) After establishing a shunt using 2 covered stents during TIPS, portography demonstrated the shunt, as well as unblocked MPV and superior mesenteric vein. However, some thrombi remained in portal vein (white arrow). CT, computed tomography.

For those with acute portal thrombosis without gastrointestinal bleeding, a percutaneous puncture was conducted to place a catheter in the portal vein for embolectomy (using 18 G guiding catheter), thrombectomy (balloon dilatation), local thrombolysis, and anticoagulant therapy. If the patient responded poorly to this approach (e.g., occurrence of gastrointestinal bleeding, as shown in Fig. 8), TIPS was performed immediately.

Twenty-one patients were implanted with bare metal stent (BMS, ev3 Endovascular, Inc., Plymouth, MN; or Cordis Corporation, Fremont, CA), 61 with covered stent (C. R. Bard, Inc., New Providence, NJ), and 16 with BMS combined covered stent. The stent diameter used in the TIPS procedure was 10 mm in 57 patients or 8 mm in 41 patients, respectively. TIPS was performed in 83 patients, whereas transcaval intrahepatic portosystemic shunt in 15 patients. If the blood flow of the shunt was not satisfactorily abundant, for thrombolysis, a pigtail catheter was indwelled at the distal end of portal vein thrombus to inject 500,000–750,000 units of urokinase daily for 1–2 days. During the intermission of thrombolysis, heparin sodium was continuously dropped through the pigtail catheter at a speed of 500 IU/h.

#### Regular postoperative observations and treatments

The patients were required to lie supinely for 24 h following TIPS and the puncture site was compressively bound up. The vital signs of each patient were closely monitored. Routine management was undertaken to prevent infection, liver dysfunction, and hepatic encephalopathy.

#### Postoperative anticoagulants

From day 2 after the procedure, low molecular weight heparin (LMWH) 5000 IU was subcutaneously injected twice daily for 5–7 days in general, followed by aspirin enteric-coated tablets (not for those with portal hypertensive gastropathy or gastric/duodenal ulcer) 300 mg once daily, which was reduced to 200 mg once daily 1 month later, and to 100 mg once daily 3 months later for long-term use. Another anticoagulant regimen was applied for those with contraindication of aspirin enteric-coated tablet: LMWH 5000 IU was subcutaneously injected twice daily for 5–7 days, followed by at least 1-year use of warfarin, while the INR was required to be maintained at 2–3.

#### Follow-up

The patients were re-examined after 1, 3, and 6 months, and then every 6 months after TIPS. Medical history and physical signs were recorded in detail. Clinical examinations such as liver and renal function tests, coagulation test, blood ammonia test, routine blood test, Color Doppler ultrasonography, barium meal radiography, and gastroscopy, as well as CT if necessary, were performed. If Color Doppler ultrasonography indicated that stenosis and occlusion of shunt had occurred, or varicosity was worsened, or the patients suffered from gastrointestinal bleeding, refractory ascites and hydrothorax, a contrast CT was performed. Meanwhile, the patients were hospitalized for the second time and the portal pressure was measured after a catheter was inserted into the portal vein through jugular vein. If blood supply of the shunt was unobstructed while portal pressure was increased or shunt was stenotic/occluded, balloon dilatation of shunt and/or stent implantation was performed. If the primary shunt was difficult to re-open or the shunt volume was insufficient, TIPS was performed for the second time to open a new shunt.

#### Statistical analysis

SPSS 17.0 software was used for data analysis. To compare means of different groups, student’s t test or one-way ANOVA was used. Paired sample t-test was used to compare the difference before and after the treatment. For categorical data, Chi-square test or Fisher’s exact test was used to compare different groups. In the between-group comparison, Bonferroni correction was used to correct the size of a test. A significant level was defined at a *P* < 0.05.

## Results

The overall successful rate of TIPS and cavernous transformation was 90.7% and 85.0%, respectively. The failure of TIPS was mainly due to 1) inability to puncture the portal vein, or to pass a catheter through either thrombus; 2) portal cavernous transformation to normal distal vessels. No patient died or presented with severe complications. Among the 98 patients, 71 (72.4%) underwent combinatory percutaneous transhepatic portal catheterization (through right hypochondriac region or subxiphoid approach) and TIPS, and 13 underwent thrombolysis therapy through the indwelling catheter at the portal vein.

Overall, 82 (83.7%) patients had completely patent shunt at 12 months after TIPS. The shunt blood flow of the remaining 16 patients (16.3%) was affected to different degrees during the procedure; among whom 9 had completely unblocked shunts at the 12th month post-procedure, 3 showed improvement, 1 showed stable disease, and 3 had developed stenosis.

Mean portal pressure was dropped from 33.08 ± 1.38 mmHg preoperatively to 20.18 ± 0.83 mmHg postoperatively (*P* < 0.001).

### Follow-up

All 98 patients were followed up for >5 years. The mean MELD score at 1-year, 2-year and 5-year after TIPS was 14.26 ± 8.07, 10.89 ± 4.11 and 11.89 ± 6.67, respectively. The bilirubin (µmol/L) level was 28.57 ± 30.12, 27.25 ± 33.51 and 27.64 ± 12.80, respectively. The ammonia level (µmol/L) was 80.46 ± 32.20, 70.15 ± 26.10 and 65. 44 ± 24.12, respectively. The ALT level (U/L) was 50.13 ± 14.28, 36.12 ± 17.33 and 28.40 ± 16.16, respectively.

### Clinical symptoms

Gastrointestinal bleeding relapsed (or newly occurred) in 39 (39.8%) patients. Refractory or newly occurred ascites or hydrothorax was present in 9 (9.2%) patients. Hepatic encephalopathy occurred in 22 (22.4%) patients pre-TIPS, and 36 (36.7%) patients during follow-up

### Secondary interventional therapy

Among the 98 patients, intervention was implemented for once in 59 (60.2%) patients, twice in 27 (27.6%) and ≥3 times in 12 (12.2%). Two patients underwent local thrombolysis + balloon angioplasty, 3 underwent pure shunt balloon angioplasty, and 34 underwent stent implantation.

### Cumulative rates of restenosis (including thrombotic relapse)

The cumulative rate of restenosis was 19.4% (19/98), 31.6% (31/98), 39.8% (39/98), 52.0% (51/98), and 61.2% (60/98) at 1, 2, 3, 4, and 5 years, respectively, following the procedure.

### Overall survival (OS)

The survival rate was 96.9% (95/98), 86.7 (85/98), 80.6% (79/98), 74.5% (73/98), and 63.3% (62/98) at 1, 2, 3, 4 and 5 years, respectively, following the procedure.

## Discussion

Despite lack of a large-scale study, the use of TIPS for portal vein thrombosis has been recently on the rise^14, 21, 23^. Most studies are focused on a mixture of partial and complete thrombosis, with a successful rate of TIPS at between 71% and 100%^14, 21, 24–28^. Nevertheless, only a few studies have focused on complete occlusion of portal vein. Perarnau *et al*. recruited 34 patients with total occlusion of portal vein and reported a successful rate of TIPS as 79%^23^. Bibao *et al*. studied 6 patients and succeeded in 6 (100%)^29^, and Walser *et al*. enrolled 15 patients and succeeded in 12 (80%)^30^. However, for either combined total and partial portal thrombosis or total thrombosis, the reported successful rate was ranged at 0–75%^23, 24, 26, 30^. One of the important reasons for the failure of TIPS lies in cavernous transformation of portal vein. The procedure-associated mortality rate varies significantly. In the 70 patients reported by Angelo^14^ *et al*., no procedure-associated death was observed. Similarly, death was reported by Perarnau^23^ *et al*. in 1 out of 34 patients (3%) or 1 out of 57 patients (1.8%) by Han^21^ *et al*. Importantly, the overall successful rate in our study was 90.7%; whereas 85% for cavernous transformation of portal vein. No case of death was noted.

In the present study, as only those patients with total occlusion of MPV were included, and 34% of patients had more than two vessels occluded, the overall thrombotic status was more severe than that in other studies. The possible reasons for achieving a higher rate of success are as follows. Firstly, stringent inclusion criteria were established for TIPS. Only those with significant clinical symptoms (gastrointestinal bleeding, refractory hydrothorax and ascites, severe dysfunction, or increased thrombus) were included. The patients have met one of the additional criteria: subacute/chronic portal vein thrombosis or cavernous transformation of portal vein (17 patients), at least one intrahepatic portal branch unblocked or existence of a relatively large branch/collateral vessel, rich in collateral vessels of MPV, relatively large collateral vessels of superior mesenteric vein or of main splenic vein; without cavernous transformation or only a few collateral vessels of portal vein (70 patients), relatively large collateral vessels of superior mesenteric venous or splenic venous trunk; a notable medical history, acute thrombosis of portal venous system (11 patients), ineffective conservative treatment or interventional therapies (embolectomy, thrombectomy, and local thrombolysis) or increased thrombus, gastrointestinal bleeding or refractory hydrothorax and ascites, or severe liver dysfunction.

Secondly, intraoperative techniques were comprehensively applied and different surgical instruments were skillfully employed accordingly. Among the 98 patients, 71 (72.4%) were treated with a combination of percutaneous transhepatic portal catheterization and TIPS. If the left or right portal branch was unblocked (48 patients), an indirect portography was performed through femoral arterial intubation followed by TIPS. If failed, percutaneous transhepatic portal catheterization puncturing through the right hypochondriac region (alternatively, subxiphoid was approached when it failed again, or when the left portal branch was unblocked) was conducted. After successful catheterization, the catheter inserted into the portal vein was used as a guideline for subsequent TIPS. If both branches of portal vein were blocked (23 patients), or intrahepatic portal vein had no branches or only collateral vessels (27 patients), percutaneous transhepatic portal catheterization was chosen. If successfully catheterized, TIPS was performed.

In some patients, no blood was drawn out after the puncture needle was inserted into the thrombus. However, a DyneCT (Siemens AG, Munich, Germany) scan confirmed that needle tip was laid inside the thrombus located in the portal branch. Therefore, the catheter was adjusted to pass through the thrombus and reach the end of the vessel. If a small catheter (Cook Medical, Bloomington, IN) encountered resistance and failed to pass through the thrombus, an 18 G puncture needle was used (9 patients), and 0.035 super-slip guide wire was inserted through the thrombus or occluded vessel, and then TIPS was performed.

If percutaneous transhepatic portal catheterization was successfully established, but TIPS failed to penetrate the portal vein, a balloon was used to dilate the portal vein and thrombus of the branch (4 patients) to create a small passage, and then portal puncture was performed for TIPS.

During TIPS procedure, all varicose veins that could be penetrated by catheter were embolized. Afterwards, when blood flow in the shunt was affected by thrombus, a 4 F pigtail catheter was deployed at the distal end of thrombus for thrombolysis.

Taken together, strict preoperative selection of patients, timely intraoperative adjustment of techniques, optimization of TIPS instrument, and postoperative intense care can remarkably elevate the successful rate of TIPS and reduce the risks of gastrointestinal bleeding as well as intra-thoracic or -abdominal hemorrhage.

In recent years, an increasing number of patients with liver cirrhosis related portal vein thrombosis has been treated with TIPS. Simultaneously, TIPS on liver cirrhosis caused thrombotic total occlusion of main portal vein has been explored by clinical researchers.

A previous study^14^ included 70 patients, only 24 (34.3%) had occluded vessels of >76% in diameter. At 12 and 24 months following the procedure, the rate of hepatic encephalopathy was 12% and 32%, respectively; the incidence of BMS restenosis was 38% and 85%, respectively; the restenosis rate in covered stent was 21% and 29%, respectively, while the overall survival rate was 89% and 81%, respectively. Perarnau^23^ *et al*. reported that in those 34 patients with thrombotic total occlusion, the 1-, 2-, and 4-year survival rate was 80%, 72%, and 55%, respectively. Another study^21^ declared that among 57 patients including 22 (38.6%) with thrombotic total occlusion, the cumulative 1- and 2-year shunt blockage rate was 21% and 32%, receptively; the incidence of hepatic encephalopathy was 25% and 27%, receptively, the cumulative 1- and 5-year variceal bleeding rate was 10% and 28%, receptively, and the cumulative 1- and 5-year survival rate was 86% and 77%, respectively.

In the current patient population, all patients had total occlusion of MPV including 27.6% with two-vessel occlusion and 6.1% with ≥3-vessel occlusion. In addition, involvement of a single vessel or ≥2 vessels was noted in 22.4% and 77.6% of patients, respectively. Main problems after TIPS included shunt restenosis (19.4%, 31.6%, 39.8%, 52.0%, and 61.2% of thrombotic relapse at 1, 2, 3, 4, and 5 years, respectively), gastrointestinal bleeding relapse (39.8%), refractory hydrothorax and ascites (9.2%), and hepatic encephalopathy (36.7%). During the follow-up (mean duration of 53.6 months), the 1-, 2-, 3-, 4-, and 5-year survival rate was 96.9%, 86.7, 80.6%, 74.5%, and 63.3%, respectively.

Thrombosis was more severe in the present study population than that in other studies; and the relapse, restenosis, and survival rates were also different from other studies. This is possibly attributed to multiple factors such as inclusion criteria, intraoperative techniques, postoperative management, and duration of follow-up.

In conclusion, TIPS has been demonstrated as an effective and safe approach in treating liver cirrhosis-related thrombotic total occlusion of MPV. Nevertheless, a careful preoperative selection of patients, implement of comprehensive techniques and intraoperative employment of appropriate instruments, regular postoperative follow-up, anticoagulant therapy, and timely secondary intervention are pivotal for ensuring the success of TIPS, reducing the risks of death and severe complications, increasing the rate of shunt unblockage, lowering the rate of clinical relapse, and prolonging the span of survival.
